# Reduced Shear Modulus and Altered Lamellar Morphology of the Outer Annulus Fibrosus in Painful Intervertebral Disc Degeneration Compared With Tissue From Non‐Surgical Controls

**DOI:** 10.1002/jsp2.70123

**Published:** 2025-10-08

**Authors:** Taylor J. Bader, Manmeet Dhiman, Lucas Lo Vercio, Jacques Bouchard, Fred Nicholls, Nathan Evaniew, Bradley Jacobs, Kenneth C. Thomas, Paul Salo, David A. Hart, Neil A. Duncan, Ganesh Swamy

**Affiliations:** ^1^ McCaig Institute for Bone and Joint Health University of Calgary Calgary Alberta Canada; ^2^ Department of Medical Sciences, Cumming School of Medicine University of Calgary Calgary Alberta Canada; ^3^ Department of Biomedical Engineering University of Calgary Calgary Alberta Canada; ^4^ Department of Surgery, Cumming School of Medicine University of Calgary Calgary Alberta Canada; ^5^ Department of Civil Engineering University of Calgary Calgary Alberta Canada

**Keywords:** annulus fibrosus, disc degeneration, intervertebral disc, shear mechanics

## Abstract

**Background:**

Stability of the spine and intervertebral disc (IVD) integrity is enabled by the highly organized fibrocartilaginous annulus fibrosus (AF). The shear properties of the AF are important in maintaining IVD integrity. AF shear mechanics in degenerative disc (DD) remain underexplored, especially in comparing minimally degenerative (non‐DD) and symptomatic DD individuals. This study measured tissue mechanical properties (AF simple shear modulus and dynamic shear properties) and examined structure (with optical coherence tomography (OCT)) in surgical DD and non‐DD control individuals.

**Methods:**

Whole AF tissue samples were collected from non‐DD donors (*N* = 13) and DD surgical individuals (*N* = 30). Two anterior outer AF (OAF) 5 mm cubes were sectioned from each sample and subjected to shear in two orientations, radial (coronal plane, G1) and circumferential (sagittal plane, G2). Tissues underwent static shear and dynamic shear protocols to a maximum of 10% shear strain. Following mechanical tests, average lamellar thickness was assessed using OCT.

**Results:**

Static shear moduli were significantly reduced for DD tissue compared to non‐DD in both the radial (G1) (non‐DD: 83.0 ± 41.3 kPa, DD: 24.1 ± 23.7 kPa) and the circumferential (G2) (non‐DD: 226.2 ± 81.9 kPa, DD: 54.0 ± 40.2 kPa) orientations (*p* < 0.05). Further dynamic mechanical alterations were detected in hysteresis, phase shift, and dynamic modulus. Shear moduli correlated negatively with lamellar thickness (G1: r_s_ = −0.63, G2: r_s_ = −0.71).

**Conclusions:**

There were significant alterations in AF shear moduli and dynamic properties in DD individuals when compared to non‐DD controls. Structural correlations highlight the role of the highly organized AF lamellar structure on shear modulus values. These findings suggest that altered AF mechanics may contribute to DD pathology and associated low back pain, warranting further investigation into structural and functional AF changes in symptomatic individuals.

## Graphical abstract

Disc degeneration (DD) results in a mechanically dysfunctional disc through progressive loss of the intervertebral disc's (IVD) highly organized structure. This study measured shear and structure properties of the outer annulus fibrosus in surgical DD and minimally degenerative (non‐DD) donor individuals.

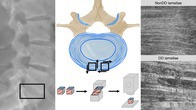

## 1 Introduction

Intervertebral discs (IVD) are comprised of a central gelatinous nucleus pulposus (NP) that is sheathed by the cartilaginous endplates and layers of fibrocartilaginous annulus fibrosus (AF). Together, these components act as the principal connection between vertebrae, providing flexibility to the spinal column and distributing loads between vertebral bodies.

By the age of 40 years old, 68% of individuals display signs of disc degeneration (DD) [1, 2], with progressive loss of structural integrity of the AF and NP [3, 4, 5]. Clinically, T2‐weighted magnetic resonance imaging (MRI) images provide a gross diagnosis of DD severity and can be graded according to the modified Pfirrmann grading system based on the reduction in distinction between AF and NP, and loss of IVD height [6]. Although many individuals can have DD and remain asymptomatic, DD is often associated with the development of low back pain (LBP) [1, 2]. LBP is a leading cause of disability worldwide and results in marked reduction in quality of life, increased disability compared to other chronic conditions, and a significant burden on healthcare costs [7, 8, 9]. Increased LBP and functional impairment can be documented preferentially in female individuals, with only limited explanation [10, 11, 12]. When conservative treatments do not reduce pain associated with the loss of IVD functionality, lumbar fusion or disc replacement surgeries can provide pain relief and functional improvement through removal of the structurally compromised disc [13].

DD results in a mechanically dysfunctional disc through remodeling of the extracellular matrix (ECM), including fibrosis, angiogenesis, innervation, and reductions in disc hydration [4, 14, 15]. In the AF, what were once highly organized lamellae of parallel collagen bundles and elastin fibers show increasingly random arrangements of collagen, irregular elastic fibers, and increased numbers of cell clusters [4, 16, 17, 18, 19, 20]. Reductions in the cross‐linking of collagen bundles through both aging and collagenase activity result in increases in lamellar thickness and reductions in total lamellar count [17, 18, 21]. Optical coherence tomography (OCT) has recently been used to assess lamellar changes associated with aging [22] and altered shear loading of AF [23, 24], providing sufficient resolution to measure individual lamellae. Alterations in lamellar organization decrease resistance to shear strains [25] and compressive forces [26], rendering the AF susceptible to further damage [27]. However, there is limited empirical data linking AF lamellar contributions to gross simple shear strain [28, 29].

Studies exploring changes in annular shear properties in DD individuals have yielded conflicting results. It has been suggested that static and dynamic shear resistance are increased with DD, although significant differences have not been documented compared to individuals with minimal amounts of degeneration (non‐DD) [29, 30]. Rather, in vivo MRI showed that DD individuals have increased lumbar motion segment rotation [31] and ex vivo functional spinal unit (FSU) torsion stiffness decreases with DD severity [32]. Similarly, studies of the circumferential tensile properties of the AF yielded a softer and weaker outer AF (OAF) correlating with DD severity in one study [33], and increased toe region stiffness in DD AF in another [34, 35]. Additionally, previously reported shear modulus values used frozen tissue [28, 29], which may lead to increased axial rotation [36]. The inconsistency of reports detailing AF shear stiffness and use of previously frozen tissue suggests that the reported values warrant replication and further detailed investigation.

DD changes lead to an abnormal stress distribution within the IVD and can induce instability of the spinal column [31, 37, 38]. Altered shear properties of the OAF could contribute to the clinical symptoms observed in DD individuals and may play a key role in the development of spinal deformities and pain commonly associated with the progression of DD. There are no prior biomechanical studies directly comparing the OAF shear properties of fresh tissue collected from symptomatic DD individuals and control non‐DD.

We hypothesized that the shear modulus would be altered in OAF tissues from DD individuals in comparison to tissue from non‐DD controls and would correlate with changes to OAF structure. We sought to measure the static and dynamic properties of fresh OAF collected from surgical DD individuals, as compared to non‐DD control tissue. The results of the study provide new insights into the relationship between the macro‐mechanical properties of DD tissue and measures of altered tissue structure and thus, clues to progression of DD and loss of tissue integrity.

## 2 Methods

### 2.1 Tissue Collection and Preparation

Non‐DD and DD tissues were obtained from the Southern Alberta Tissue Donation Program and patients undergoing spinal surgery at the Foothills Medical Center in Calgary, respectively (Ethics ID: REB 18‐1308). Non‐DD donors were identified by the donor coordinator, and third‐party consent was acquired through surrogate or parental authorization. For DD individuals, consent was obtained preoperatively.

Computed tomography (CT) images of the abdomen were used for all consented non‐DD individuals to confirm no visible olisthesis or coronal translation, and were assessed for degenerative changes using CT images and a radiographic grading system [39, 40]. DD individuals had available radiographs and MRI images, and degeneration was graded using the modified Pfirrmann grading system [6].

**FIGURE 1:**
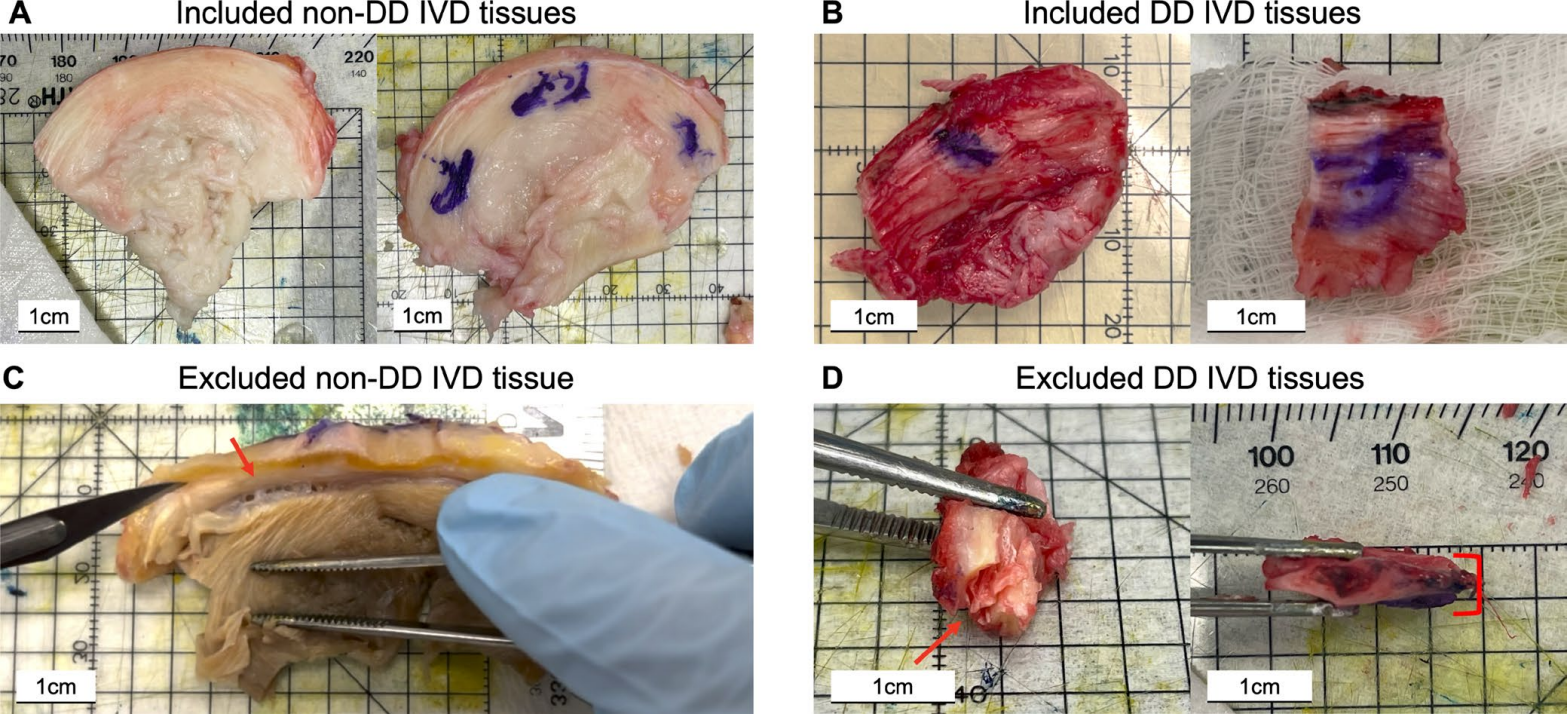
Included and excluded portions of disc collected. (A) Non‐degenerative (non‐DD) intervertebral disc (IVD) tissue collected from tissue organ donors. Tissue labeled to maintain orientation. (B) Degenerative disc (DD) IVD tissue collected from an anterior approach interbody fusion or arthroplasty. (C) Excluded non‐DD tissue. Red arrow highlighting large annular fissure. (D) Examples of excluded DD tissue. Red arrow highlighting large annular fissure. Red bracket highlighting disc height below 5 mm.

For non‐DD donor tissue collection, anterior portions of the L4‐5 and L5‐S1 discs were removed within 2 h postmortem following laparotomy for abdominal organ retrieval (Figure 1A). DD tissues were obtained intraoperatively from L4‐5 and L5‐S1 anterior lumbar surgeries, including anterior lumbar fusions and lumbar total disc arthroplasty (TDA) (Figure 1B).

Superior‐ and anterior‐surfaces of collected AF tissues were marked using tissue‐marking dyes to maintain orientation throughout processing. Tissues were immediately wrapped in a Telfa gauze pad soaked in sterile saline. The tissues were transiently stored in small water‐tight containers and testing was initiated within 45 min of dissection or surgery. Tissue with annular fissures, large blood vessels, or axial height less than 5 mm was excluded from the study (Figure 1C,D).

**FIGURE 2:**
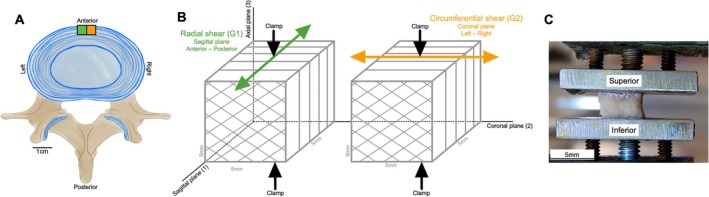
Tissue orientation and mounting. (A) Outer annulus fibrosus tissues were all collected from the anterior portion of the disc and sectioned into two 5 mm cubes (green and orange boxes). (B) One cube was sheared in the G1 plane (radial, green line), and another in the G2 plane (circumferential, orange line). (C) 5 mm tissue cubes were mounted in the axial plane (3) with their superior and inferior surfaces adhered to adjacent clamps.

Tissues were dissected into two 5 mm cubes of the OAF (Figure 2A). Subsequently, the cubes of tissue were mounted and subjected to shear in two planes: sagittal (anterior–posterior, radial, G1) and coronal (left–right, circumferential, G2) (Figure 2B). These orientations were chosen to mimic the directions of shear that could occur between two adjacent vertebrae and to consider in‐plane (G2) and out‐of‐plane (G1) lamellae properties. The 5 mm tissue cubes were tested at the same time on two test frames, a Bose 3230 and a Bose 3220 test frame (TA Instruments, New Castle, DE) with 10 N Bose load cells (±0.01 N). The superior and inferior surfaces of the tissue were adhered to flat, parallel custom‐manufactured aluminum and acrylic clamps using cyanoacrylate gel (Gorilla Superglue; The Gorilla Glue Company, Ohio, USA) (Figure 2C). The clamps were tightened to a 4.5 mm gap between the clamps (approximately a 10% compressive pre‐strain). Confirmation of tissue adherence to the clamps was performed using video recordings and a live preview using a tripod‐mounted GoPro camera (GoPro Hero 7) (Video S1). After mounting, tissues were kept at room temperature (21°C) and received constant 100% humidity (Hanna HI 9065) [41].

### 2.2 Biomechanical Testing and Analyses

Each OAF tissue cube underwent a total of four mechanical tests separated into two sets. The first set consisted of a Dynamic Mechanical Analysis (DMA) to examine rate‐dependent shear, followed by a Static Shear Analysis (SSA) to examine strain relaxation, both at 10% shear strain.

**FIGURE 3:**
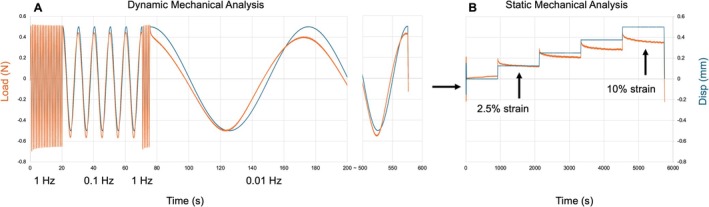
Mechanical protocols at 10% shear (±0.5 mm) strain under displacement control (mm) (blue line) and measured load (N) (orange lines). (A) Dynamic Mechanical Analysis (DMA) included preconditioning with 20 cycles at 1 Hz. Subsequently, 5 cycles at 0.1, 1, and 0.01 Hz were performed in alternating sequence. (B) Static Shear Analysis (SSA) included tissues remaining at rest for 15 min before being sheared at 2.5% strain intervals held for 20 min. Intervals were repeated until 10% total strain was applied. After 10% tests were complete, 40% shear strain DMA (±2 mm) and SSA (10% strain intervals held for 5 min) protocols were performed.

Prior to beginning the protocols, each sample was subjected to ±0.5 mm to evaluate positive and negative loads. A position that resulted in roughly equal positive and negative loads (±0.1 N) was set as the resting displacement (displacement = 0). The DMA protocol was initiated with 20 cycles of preconditioning to 10% strain (±0.5 mm) at 1 Hz followed by five cycles each at 0.01, 0.1, and 1 Hz applied to the cubes in alternating sequence (Figure 3A) [29, 42, 43, 44, 45]. Using a custom‐developed MATLAB script, data from each frequency was split and analyzed. The final period of each frequency was used for analysis to limit results from previous frequencies. Hysteresis was measured from the area under the curve between the loading and unloading waveforms. The tangent of the phase shift (δ) was calculated by applying a Fast Fourier Transform (FFT), normalization, and direct current (DC) balance to the load and displacement data [42, 43]. Loss modulus (G′) (Equation 1), storage modulus (G″) (Equation 2), and dynamic shear modulus (|G*|) (Equation 3) were calculated using shear stress (σ), shear strain (ε), and δ.

(1) $$G^{\prime }=\frac{\sigma }{\varepsilon }\sin\left(\delta \right)$$

(2) $$G^{\prime \prime }=\frac{\sigma }{\varepsilon }\cos\left(\delta \right)$$

(3) $$\left|G^{*}\right|=\sqrt{{\left(G^{\prime }\right)}^{2}+{\left(G^{\prime \prime }\right)}^{2}}$$

The SSA protocol was initiated with 5 cycles of preconditioning at 2.5% strain (±0.125 mm) followed by 15 min of the tissue at rest (Figure 3B). Unidirectional step displacement of the clamps was increased by 2.5% strain every 20 min, repeated four times, resulting in net strains of 2.5%, 5%, 7.5%, and 10%. Each step was performed at a rate of 0.2 mm/s in concordance with previous studies [28, 46]. A relaxation time of 20 min was chosen following pilot experiments at various time intervals to determine cessation of tissue relaxation and to replicate a similar protocol design reported previously [28]. The equilibrium stress determined at each strain increment was calculated using the increased load value of each 20‐min interval relative to the load at the end of the 15 min at rest. Shear modulus was calculated as the slope of the line of best fit between each equilibrium stress value at 2.5% strain increments up to 10%. Stress relaxation was calculated by subtracting the relaxed stress from the peak stress at each assessment interval.

The second set of tests included an additional DMA and SSA protocol, this time at 40% shear strain. As 10% strain generally did not result in tissue leaving the neutral zone (NZ) in most samples, 40% strain DMA (±2.0 mm) was performed as per Smit et al. to measure the trilinear fit for NZ stiffness, compression zone (CZ) stiffness, and tension zone (TZ) stiffness, in addition to NZ length (Figure S1) [47].

A shear strain of 40% is in excess of physiological values and potentially results in damage to the tissue [24, 48]. However, a series of pilot experiments showed that some DD tissue specimens displayed an order of magnitude of reduction in shear modulus. To ensure that differences in tissue stiffness were captured, tissues were subjected to a 40% strain SSA protocol (net strain of 10%, 20%, 30%, and 40%). At 40% strain, SSA relaxation times were reduced to 5 min, likely resulting in elevated shear modulus values, as the tissue had not yet fully relaxed. This change was made to minimize the testing time (totaling 135 min) and to limit the tissue's exposure to destructive shear strain environments.

### 2.3 Optical Coherence Tomography Analyses

Immediately after mechanical tests were completed, the AF cubes were removed from the mount for OCT imaging. Approximately 1 mm of tissue from each end of the cube was removed, as cyanoacrylate had been used on the surfaces to clamp tissues, leaving a final dimension of 5 mm × 5 mm × 3 mm in the coronal, sagittal, and axial planes, respectively.

OCT analysis was performed on a Thorlabs Callisto System with a 900 nm rigid scanner (OCTG‐900). A Thorlabs Telesto‐II spectral domain OCT system was used with an OCT‐LK4 Scan Lens and LSM04 general purpose imaging objective. Nominal wavelength was 1310 nm. Sensitivity was set to medium (48 kHz), and A‐scan averages were three. Overall scan field of view and resolution were variable. Fixed pixel sizes of 10.23 μm in the x and y direction and 2.48 μm in the z direction were used. Acquisition time totalled 47 s. A maximum depth of 2.54 mm was recorded, and the field of view captured the entire cube. The resulting scan was saved as 1024 cross‐sectional slices. From this stack, the most superior section capturing the entire surface area of the tissue was used for further analysis.

**FIGURE 4:**
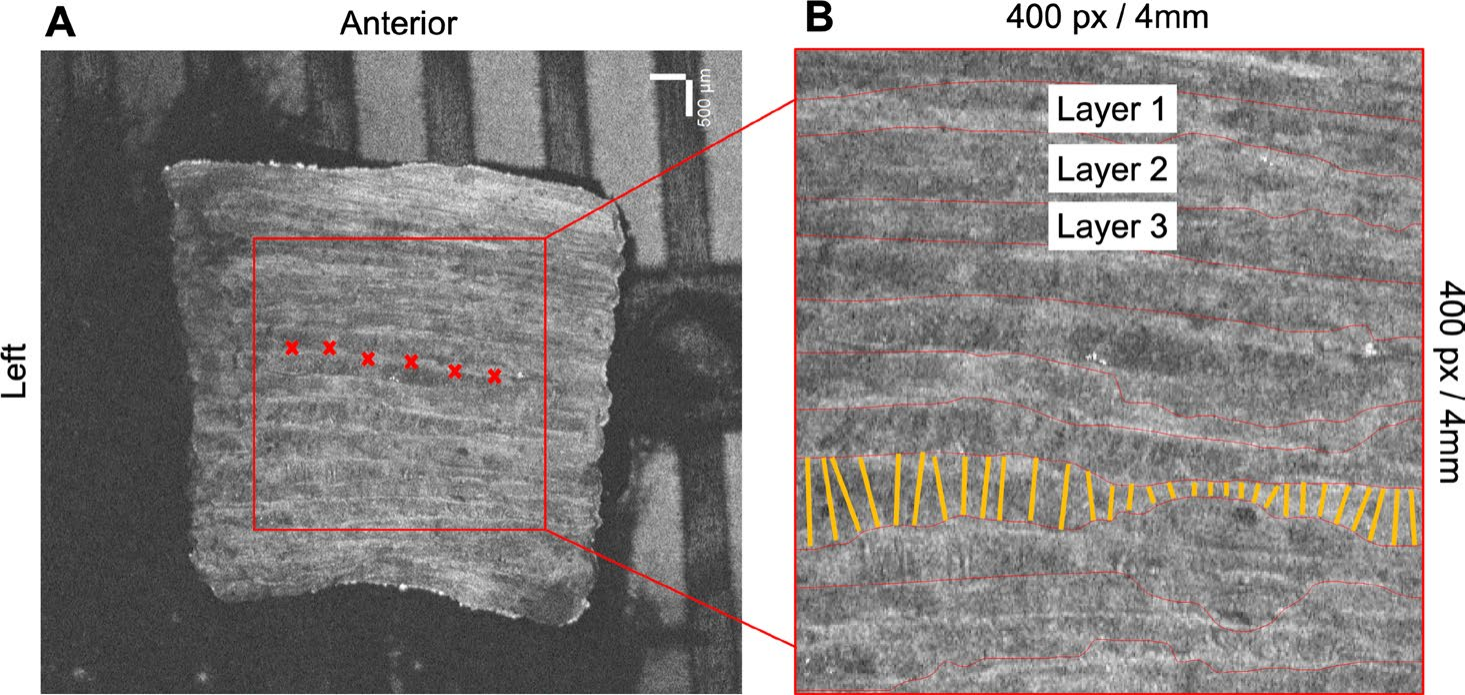
Optical coherence tomography imaging and lamellae identified. (A) Raw 2D layer of an annulus fibrosus tissue. The inner 4 mm × 4 mm area was cropped and used for layer analysis (red box). Layer identification was initiated by 10–20 manually applied markers to each lamellae border (red x). (B) Analyzed 400‐ × 400‐pixel image. Layers included in analysis had two labeled borders and spanned the entire image (e.g., layer 1, layer 2, layer 3). Lamellar thickness measurements are visualized as perpendicular lines between two segments (yellow lines).

To measure lamellar thickness, the edges of the images were cropped to limit empty space. Acquired images were cropped to a standardized central 400‐ × 400‐pixel image (4 mm × 4 mm) (Figure 4A). To characterize the border between two lamellae, a 2‐class Random Forest (RF) was trained with 100 trees, evaluating five features in each split of the trees [49, 50]. The features computed [51] for each pixel were obtained from: the original image; detail‐preserving anisotropic diffusion (DPAD) of the image using 1000 and 5000 iterations; the z‐score of the median filtered image with windows 5 × 5, 15 × 15, 31 × 31, 100 × 3 and 3 × 100; the z‐score of the Haralick's texture features (angular second moment, entropy, sum of squares) of the DPAD‐filtered image using a 7 × 7 window, and in the original image using a 7 × 21 window with d = −1 and d = −2 in the vertical direction; the z‐score of the speckle index in 5 × 5, 11 × 11, 101 × 3 and 3 × 101 windows. Four images were used for training, where each layer was annotated as bright or dark.

After an image was characterized at the pixel level using the RF, the segmentation was used as an input to a modified version of Cimtool [52]. The interface of this tool allows the selection of multiple points in the transition between lamellae for initialization. Manually initialized lines were chosen by blinded members of the study team who were tasked with defining lamellae boundaries that spanned the entire 400 pixel/4 mm of the image. Lamellar thickness measurements calculated from the pixel count between perpendicular nodes from each segment were computed using methods from a previous study (Figure 4B) [52]. Pixel measurements were converted to distance in mm.

### 2.4 Statistical Analysis

All statistical tests were performed in R (RStudio and Virtual Studio Code). Statistical significance was set at *α* = 0.05. Data normality was assessed using the Shapiro–Wilk test. For normally distributed data (*p* > 0.05), repeated‐measures ANOVA was used for within‐group comparisons, and Welch's t‐test was used for between‐group and post hoc comparisons. For non‐normally distributed data (*p* < 0.05), the Friedman test was used for within‐comparisons, and the Mann–Whitney U test was used for between‐group and post hoc comparisons. Bonferroni corrections were applied for multiple comparisons. Spearman's rank correlation was used to assess relationships between Modified Pfirrmann Grade, average lamellar thickness, and shear moduli.

To account for potential deviations from normality and false positives, all comparisons were reanalyzed using parametric and non‐parametric methods, confirming that statistical significance remained unchanged.

## 3 Results

OAF tissues were collected from a total of 8 non‐DD and 31 DD individuals (Table 1). One non‐DD individual was excluded due to a visible annular fissure (Figure 1C), and 6 DD individuals were excluded due to the collected tissue being delaminated or too thin (Figure 1D). When available, multiple disc levels from the same individual were included, and each disc was graded for degeneration following suggested study design practices [41]. Due to significant differences between groups in age, an age‐matched cohort was generated using a propensity matching algorithm (matchit) (Table S1) [53].

**TABLE 1:** Demographics of the individuals included in the study.

		Non‐DD	DD
Individuals	Included individuals	7	25
	Age (years)*	37.6 ± 6.6	45.7 ± 10.9
	Sex (Female/Male)	3/4	15/10
	BMI (kg/m^2^)	—	26.1 ± 3.5
Discs	Total included discs (L4‐5/L5‐S1)	13 (7/6)	30 (11/19)
	Modified Pfirrmann grade (1–8)	—	6.5 ± 1.6
	Radiographic degeneration grade (0–8)	0.25 ± 0.45	—
Cubes	SSA OAF cubes tested (G1/G2)	26 (13/13)	52 (28/24)
	DMA OAF cubes tested (G1/G2)	19 (13/6)	45 (22/23)
	Lamellae measurements	13	24

*Note:* Total number of individuals, discs collected, and outer annulus fibrosus (OAF) tissue in each condition. Cubes tested in Static Shear Analysis (SSA), Dynamic Mechanical Analysis (DMA), and lamellae measurements. (—) denotes data that is unavailable. (*) denotes statistically significant differences (*p* < 0.05) between groups.

### 3.1 Biomechanical Analysis

**FIGURE 5:**
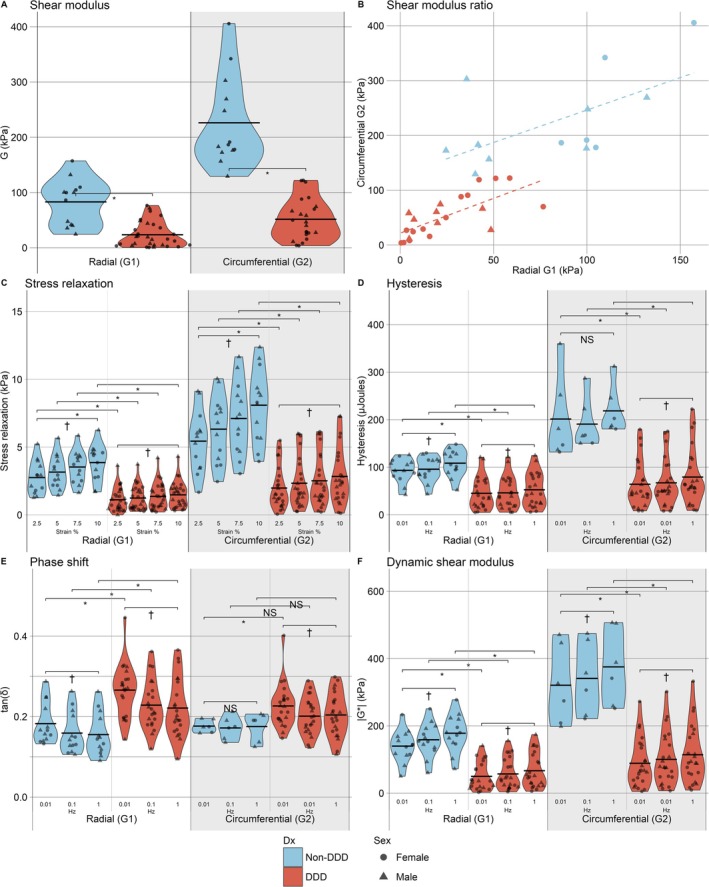
Static mechanical analysis (SSA) and dynamic biomechanical results for both G1 (radial) and G2 (circumferential) orientations. (A) Shear modulus (G) (kPa). (B) Shear modulus from adjacent cubes from the same disc. Average ratio of G2 to G1 shear moduli presented as a dashed line. (C) Stress relaxation (kPa) measured at each shear strain percent (strain %). (D) Hysteresis (μJoules) measured at each frequency (Hz). (E) Phase shift (tan (δ)) measured at each frequency (Hz). (F) Dynamic shear modulus (kPa) at each frequency (Hz). (*) denotes statistically significant differences (*p* < 0.05) in values between groups. (^†^) denotes statistically significant differences (*p* < 0.05) in values between strain percent or frequency within group and orientation. (ns) denotes no significant difference in comparison. Mean denoted with a horizontal bar. Individual disc presented as a data point labeled based on sex. Color of volcano plot denotes diagnosis (Dx) group.

For SSA measurements, DD tissue had significantly lower shear moduli than non‐DD tissue in both orientations (*p* < 0.05) (Figure 5A). The ratio of the G2 (circumferential) shear modulus to the G1 (radial) shear modulus was not significantly different between non‐DD (2.7 times greater) and DD tissues (2.1 times greater) (Figure 5B) (*p* = 0.55). Modified Pfirrmann grade score was correlated with G1 (r_S_ = −0.55, *p* = 0.003) and G2 (r_S_ = −0.60, *p* = 0.003) DD tissue shear moduli. There were no correlations between age or BMI and DD tissue G1 shear modulus (r_S_ = 0.26 (*p* = 0.17), r_S_ = 0.16 (*p* = 0.43), respectively) or G2 shear modulus (r_S_ = 0.23 (*p* = 0.28), r_S_ = 0.37 (*p* = 0.09), respectively). Similarly, there were weak correlations between age and non‐DD tissue G1 shear modulus (r_S_ = 0.25 (*p* = 0.41)) or G2 shear modulus (r_S_ = 0.44 (*p* = 0.13)). Stress relaxation was significantly decreased between the non‐DD and DD groups (*p* < 0.05) (Figure 5C).

For DMA measurements, 7 of 13 non‐DD tissues in the G2 group were excluded from analysis due to data filtering applied from a new 10 N Bose load cell. Filtering data during testing results in inaccurate phase shift and hysteresis waveforms. Results for DMA measurements in the G2 orientation represent 23 DD tissue and 6 non‐DD.

G1 and G2 hysteresis was reduced in DD compared to non‐DD tissue across all comparisons (*p* < 0.05) (Figure 5D). G1 phase shift was statistically increased in DD tissue compared to non‐DD tissue (*p* < 0.05), and G2 phase shift displayed similar but less significant trends (0.01 Hz = *p* < 0.05, 0.1 Hz and 1 Hz = *p* > 0.05) (Figure 5E). G1 and G2 dynamic shear modulus in non‐DD tissues was increased compared to DD tissue (*p* < 0.05) (Figure 5F). Both static and dynamic biomechanical results from 10% shear strain, including comparisons between orientations, are summarized in Table 2. Normality and detailed statistical results are summarized in Table S2.

**TABLE 2:** Summary of mechanical data collected from degenerative disc (DD) and non‐degenerative (non‐DD) individuals.

		Radial (G1)		Circ (G2)	
		Non‐DD	DD	Non‐DD	DD
Shear modulus (kPa)	10% strain	83.0 ± 41.3*	23.7 ± 22.1	226.2 ± 81.9* ^#^	52.2 ± 36.8^#^
Stress relaxation (kPa)	2.5% strain	2.8 ± 1.2* ^†^	1.1 ± 0.9	5.4 ± 2.2* ^# †^	2.0 ± 1.5^#^
	5% strain	3.2 ± 1.2* ^† a^	1.2 ± 0.9	6.3 ± 2.4* ^# † a^	2.3 ± 1.9^#^
	7.5% strain	3.5 ± 1.2* ^† a b^	1.4 ± 1.0	7.1 ± 2.7* ^# † a b^	2.5 ± 1.9^#^
	10% strain	3.9 ± 1.3* ^† a b c^	1.5 ± 0.9	8.1 ± 2.7* ^# † a b c^	2.8 ± 2.1^#^
Hysteresis (μJoules)	0.01 Hz	93.5 ± 25.2* ^†^	45.2 ± 33.7	201.5 ± 89.5* ^#^	62.9 ± 48.6^†^
	0.1 Hz	95.9 ± 26.0* ^†^	46.2 ± 33.1^†^	190.7 ± 54.2* ^#^	65.8 ± 49.0^† a^
	1 Hz	108.6 ± 27.8* ^† a b^	52.6 ± 35.2^† a b^	218.6 ± 51.8* ^#^	77.7 ± 58.8^† a b^
Tan (δ)	0.01 Hz	0.18 ± 0.05^† b c^	0.27 ± 0.06* ^† b c^	0.18 ± 0.02	0.23 ± 0.05* ^† b c^
	0.1 Hz	0.16 ± 0.05^†^	0.23 ± 0.06* ^†^	0.17 ± 0.03	0.20 ± 0.04^†^
	1 Hz	0.16 ± 0.05^†^	0.22 ± 0.07* ^†^	0.18 ± 0.04	0.20 ± 0.05^†^
|G*| (kPa)	0.01 Hz	139.8 ± 46.8* ^†^	49.8 ± 41.5^†^	320.7 ± 116.8* ^# †^	86.9 ± 70.2^†^
	0.1 Hz	158.7 ± 50.1* ^† a^	57.1 ± 45.9^† a^	340.9 ± 108.8* ^# †^	98.2 ± 76.6^† a^
	1 Hz	178.1 ± 54.9* ^† a b^	66.6 ± 51.5^† a b^	375.3 ± 113.3* ^# †^	111.8 ± 84.1^† a b^

*Note:* Values are presented as mean ± standard deviation. (*) denotes statistically significant increases (*p* < 0.05) between non‐DD and DD within the same orientation. (#) denotes significant increases (*p* < 0.05) between orientations within the same diagnostic group. (^†^) denotes statistically significant differences (*p* < 0.05) between strain percent or frequency within the same group and orientation. (a b c) denotes statistically significant increases (*p* < 0.05) compared to ranked values in increasing order, as determined by post hoc tests.

Results from age‐matched comparisons exhibited similar results and are summarized in Table S3. Sex comparisons resulted in no significant differences but suggested a trend toward increased stiffness in female tissues (summarized in Table S4). Results from 40% shear strain tests followed similar trends to the 10% shear strain results (Table S5). Additional analysis of the NZ found length did not display differences between groups or frequencies, while NZ stiffness, CZ stiffness, and TZ stiffness were reduced in DD compared to non‐DD tissue (*p* < 0.05).

### 3.2 OCT Analysis

**FIGURE 6:**
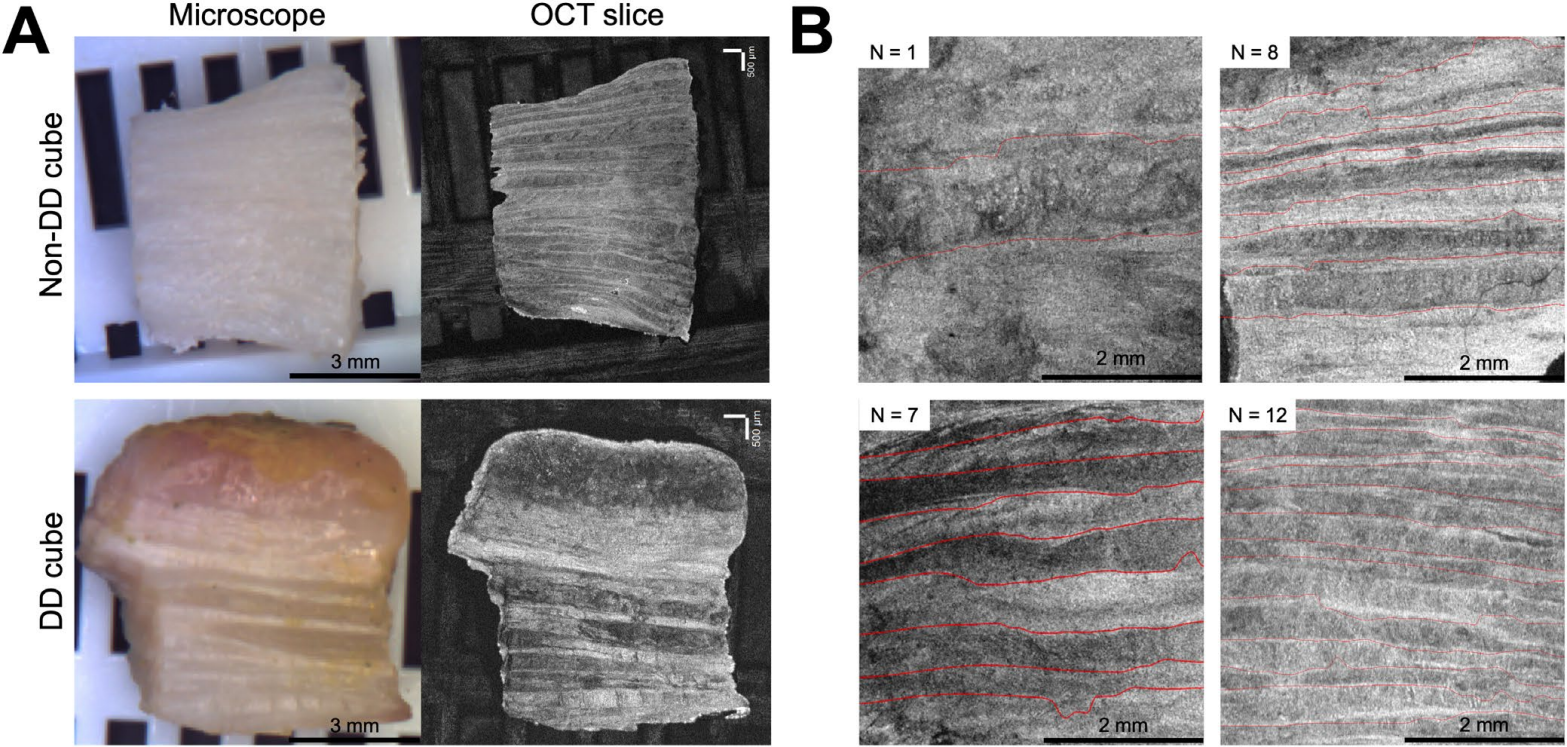
Optical coherence tomography example images. (A) Raw microscope (left) and optical coherence tomography (OCT) (right) captured images. (B) Visualization of lamellar thickness of a centered 4 mm × 4 mm OCT image. Red lines indicate separation between adjacent lamellae generated after labels were placed along the lamellar border.

**FIGURE 7:**
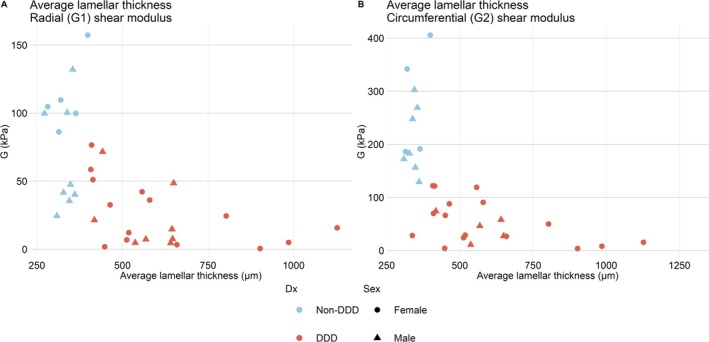
Average lamellar thickness from one 4 mm × 4 mm tissue scanned using optical coherence tomography (OCT) and correlations with 10% strain shear moduli from the same 5 mm cube. (A) Lamellar thickness correlated with G1 shear modulus. (B) Average lamellar thickness correlated with G2 shear modulus. Individuals labeled based on sex. Color of data point denotes diagnosis (Dx) group.

OCT scans provided better distinction of adjacent lamellae compared to microscope images, with examples of each provided in Figure 6A. Examples of segmentation of adjacent lamellae are provided in Figure 6B. Within the analyzed 4 mm × 4 mm OAF section, DD tissue had significantly increased average lamellar thickness compared to non‐DD tissue (*p* < 0.05) (Table 3). OCT lamellar thickness was significantly correlated with both G1 and G2 shear moduli (*p* < 0.05) (Figure 7A,B, Table 4).

**TABLE 3:** Optical coherence tomography (OCT) results.

	Non‐DD	DD
Average lamellar thickness (μm)	324.67 ± 26.76	600.23 ± 198.58*

*Note:* Average lamellar thickness of degenerative disc (DD) and non‐degenerative (non‐DD) 4 mm × 4 mm OCT images. Values are presented as mean ± standard deviation. (*) denotes statistically significant increases (*p* < 0.05) between non‐DD and DD diagnostic groups.

**TABLE 4:** Summary of Spearman's rank correlation coefficient (r_s_) between optical coherence tomography (OCT) results with radial (G1) and circumferential (G2) shear moduli.

	Radial (G1)	Circumferential (G2)
Average lamellar thickness (r_s_)	−0.63^†^	−0.71^†^

*Note:* (^†^) denotes statistically significant (*p* < 0.05) Spearman's rank correlation coefficient.

## 4 Discussion

To our knowledge, this is the largest study of experimental AF shear properties [30] and the first to compare the shear properties of OAF tissue from symptomatic DD individuals undergoing spinal fusion or arthroplasty to donor non‐DD individuals. The substantial alterations in shear behavior and structural properties in DD individuals underscore the importance of studying symptomatic tissue in addition to young organ donor tissue. Including a large cohort of young non‐DD organ donors also provides a novel and robust baseline for understanding minimally degenerative AF biomechanics. Together, these results demonstrate that AF structural and functional deterioration may contribute significantly to spinal instability and LBP that qualifies individuals for surgical intervention.

### 4.1 Reduced Shear Moduli in DD Individuals

DD severity, as graded by MRI, correlates with LBP [2] and biomechanical changes of the AF [33]; however, some individuals with visibly severe DD may remain asymptomatic [1, 2, 3]. This study aimed to characterize the functional properties of OAF tissue in symptomatic DD individuals and control non‐DD individuals. This study found reductions in static shear moduli, stress relaxation, hysteresis, and dynamic shear modulus in DD tissue compared to non‐DD control tissue.

A key consideration in interpreting these findings is the distinction between DD tissue from symptomatic surgical patients and DD donor tissue used in previous studies [29, 30, 32, 33, 34, 35]. Donor tissue, even if moderately to severely degenerated, may come from individuals who were asymptomatic. The present study, in contrast, includes individuals undergoing surgery for operable LBP or lumbar radiculopathy, potentially representing a subset of DD individuals with more severe mechanical instability. The observed reductions in shear moduli could therefore reflect biomechanical changes specific to symptomatic DD, reinforcing the importance of studying pain‐associated surgically acquired tissue in addition to cadaveric donor tissue.

Prior clinical studies reported an increased torsional range of motion in IVDs of individuals with severe DD and associated pain [31, 54]. A reduction in shear modulus may intuitively contribute to the elaboration of pain, which may be resolved through spinal fusion [31]. By including symptomatic surgical patients and minimally degenerative donor individuals, this study captures a wide spectrum of pathology. However, because this study is cross‐sectional and reflects a single point in time, the tissues assessed may represent different stages or subtypes of the degenerative process [55, 56].

Furthermore, Dhiman et al. reported reduced interlamellar mechanical properties in degenerated samples compared to the non‐degenerated samples [57]. Since bulk radial shear involves interlamellar deformation [58], these findings further support reduced radial shear resistance in the OAF of degenerated tissue.

### 4.2 Differences in Shear Moduli Between Studies

Shear moduli values reported in the present study were somewhat different from previous experimental simple shear [28] and torsion [29] reports (Table 5). Interestingly, the present results align more closely with recent finite element model (FEM) simulations, which incorporated human and bovine experimental data and similar testing parameters to the present and prior experimental studies [62]. Discrepancies in shear moduli in different reports may be the result of differences in tissue collection and testing methods.

**TABLE 5:** Comparison of human experimental annulus fibrosus shear moduli.

Author	Year	Tissue	Pre‐strain	Protocol	Dx	Age	Shear modulus (kPa)		
							G1	G2	G3
Fujita et al. [28]	2000	Cube (3.2 mm^3^)	10%	Simple shear	Non‐DD	~64 ± 25	40 (20)	60 (30)	20 (10)
Iatridis et al. [29]	1999	Cylinder (5 mm dia. × 1.7 mm h.)	35 kPa	Torsion	Non‐DD	—	—	110	—
					DD	—	—	130	—
Present study	2025	Cube (5 mm^3^)	10%	Simple shear	Non‐DD	37 ± 7	83	226	—
					DD	46 ± 11	24	54	—

*Note:* Shear strain orientations categorized as G1 (radial), G2 (circumferential), G3 (axial). Due to Poisson's ratio depending on tissue degeneration [59, 60] and potentially having negative values [61], the present study reported raw shear modulus values for simple shear without conversion for a 0.5 Poisson's ratio as done in Fujita et al. to estimate a true shear modulus. Values within brackets note shear moduli values with removal of Poisson's ratio conversion. (—) denotes no reported information in that category.

The present study included symptomatic DD individuals undergoing spinal fusion or arthroplasty as opposed to potentially asymptomatic DD donor tissue. For example, Iatridis et al. categorized DD severity of donor tissue based on Thompson grades [63], grouping grades I–II as “non‐DD” and grades III–V as “DD”. The present study had tissue collected from individuals with comparable moderate to severe degeneration (Modified Pfirrmann Grades 4–8, corresponding to Thompson grades III–V) [6, 65, 66]. Opposing trends in shear modulus may reflect these discrepancies in comparison groups.

Variability in shear modulus between this and previous studies may also result from individual ages. Previous studies only reported the age of half of their included individuals (aged 36, 71, and 86) [28], or did not report age [29]. Results from the present study likely represent younger, middle‐aged individuals.

Comparable studies performed tests with tissue submerged in saline and protease inhibitor baths [28, 29]. However, prolonged submersion (> 1 h) can induce tissue swelling, potentially altering tissue mechanics [41]. To minimize this effect, the present study used misting of the testing chamber to maintain 100% humidity instead of immersion in saline, which may have influenced shear modulus measurements. A similar trend was observed by Iatridis et al. when comparing their shear modulus measured in a saline bath to previous studies using a misting protocol [29].

Finally, and importantly, tissue in the present study was tested fresh, within 45 min of excision, whereas prior studies used tissue samples that had been frozen at various temperatures for variable periods of time. Although freezing is a common practice in biomechanics research [41], multiple freeze–thaw cycles can contribute to an increased range of motion of the spinal unit [36] and thus may contribute to differences in reported static and dynamic shear properties.

### 4.3 Lamellae Measurements Using OCT

Lamellae organization likely deteriorates with age and DD due to reductions in collagen fibers turnover and increased collagenase activity [21, 67]. In a study using ultrasonography, AF lamellar thickness in vivo was 230 ± 92 μm in adolescent individuals (10–16 years‐old) [22]. In another study, anterior AF lamellar thickness measured manually ex vivo increased from 180 ± 20 μm to 420 ± 60 μm when comparing young (18–29 years old) and aged individuals (53–76 years‐old) [17].

In the present study, measurements of lamellar thickness were performed ex vivo using OCT. Non‐DD individuals were middle‐aged (38 ± 7) and presented values between young and aged individuals in previous studies (336 ± 27 μm). DD individuals had lamellar thickness values exceeding previous studies (600 ± 199 μm), despite being younger (46 ± 11) than their aged counterparts. Lamellar thickness was not significantly correlated with age; however, it was different between non‐DD and DD individuals. Both aging and pathological disassembly of the tight collagen bundles and highly organized matrix may contribute to the development of weakness in the AF to shear stresses [39].

Lamellar thickness was measured within a transverse 4 mm × 4 mm area from the same 5 mm OAF cube used for mechanical testing. Functional measurements significantly correlated with both increased lamellar thicknesses. Lamellar organization is vital to the function of the IVD, with reductions in organization leading to reductions in AF shear and compression stiffness [25, 26, 27]. The relationship between lamellae remodeling and shear properties of the AF likely results in further reductions in each parameter, exacerbating DD and contributing to further structural failure past what is seen in aged individuals [27].

## 5 Limitations

Control tissue was defined as ‘non‐DD’ as it likely does not represent normal, as the tissue was taken from organ donors with an average age where a majority of individuals display signs of DD [1]. Additional cofactors in the development of DD, such as injury, genetics, or smoking, were also not controlled for [68, 69, 70]. However, age was an important factor in this study, so the inclusion of similarly aged individuals in both cohorts enabled an age‐matched comparison.

Testing fresh tissue depends on the native size of the tissue and the approach used by the surgeon to excise a large piece of tissue *en bloc*. As such, some tissues were deemed untestable due to overt defects such as annular delamination or had disc height below the inclusion criteria for this study (< 5 mm). Only anterior approach surgeries provided sufficient tissue for multiplane tests; thus, data from this study reflect only anterior portions of the disc. FSU torsion studies include contributions from multiple disc regions, whereas this study focused specifically on the anterior OAF, which is stiffer than the posterolateral AF [28, 71]. This regional variation should be considered when comparing shear properties across studies.

Heterogeneity in the AF is expected across the entire IVD [28, 71]. Although pilot data found limited variations between the majority of adjacent 5 mm cubes (±4.9 kPa, Table S6), cubes sectioned from the excluded non‐DD tissue (Figure 1C) displayed varying shear moduli based on proximity to the annular fissure (Far: G1 = 138 kPa, G2 = 128 kPa; Near: G1 = 25 kPa; G2 = 39 kPa).

Additionally, the applied 10% pre‐strain compression to non‐DD and DD tissues in this study may not represent axial deformation in vivo. Studies using FSU have reported increased relative axial deformation when DD IVD is compressed [71]. In comparison, previous studies found that different amounts of pre‐strain compression resulted in limited changes in G2 shear modulus in isolated OAF tissue [28, 29].

Lamellar thickness only includes lamellae within the 5 mm OAF cube and does not account for total lamellae changes. OCT imaging was unable to distinguish between lamellae and inter‐lamellar matrix, potentially amalgamating measurements of both variables. Additional expansion of these techniques should also include the qualification of lamellar density changes across larger sections of AF or the entire IVD. With further validation through histology or micro‐computed tomography, future studies should consider OCT scanning as a fast non‐destructive way of quantifying AF structure.

## 6 Conclusions and Future Directions

In this study, we investigated the shear properties and lamellae organization of the anterior OAF. DD tissue had decreased shear moduli compared to non‐DD tissue in both G1 and G2 orientations. Shear moduli negatively correlated with average lamellar thickness from the same 5 mm OAF section. Lamellar thickness was significantly increased in DD OAF tissue.

Reductions in the shear moduli in symptomatic DD OAF is a key understanding in the progression and potential treatment of this painful condition. Further studies exploring shear properties of the AF should also explore the comparison of aged asymptomatic individuals, spinal deformities including scoliosis and spondylolisthesis, and changes across the entire disc.

These large increases in lamellar thickness highlight their potential role in diagnosing AF instability using similar techniques to Langlais et al. [22] and further define changes in AF lamellar organization in symptomatic DD individuals. These trends may be further explored through more detailed structural analysis, including further histology analysis and validation of OCT scans to ensure accurate measurements. Lastly, including the quantification of tissue components and remodeling processes may further explain reductions in shear modulus in symptomatic DD individuals.

## Author Contributions

Conceptualization: G.S., D.A.H., N.A.D., P.S., T.J.B.; Methodology: G.S., D.A.H., N.A.D., P.S., T.J.B.; Surgical tissue collection: G.S., P.S., F.N., B.J., N.E., K.C.T.; Data analysis: T.J.B., L.L.V., M.D., N.E.; Manuscript preparation: T.J.B., M.D., L.L.V., J.B., F.N., N.E., B.J., K.C.T., P.S., D.A.H., N.A.D., G.S.

## Conflicts of Interest

The authors declare no conflicts of interest.

## Supporting information


**Figure S1:** Dynamic Mechanical Analysis (DMA) at 40% shear strain. (A) Neutral zone (NZ) calculation: To separate the curve into a compressive shear zone (CZ), tensile shear zone (TZ), and NZ, the borders of the NZ were determined using the maximum and minimum of the second derivative, which would note the change in shear compliance. A linear line of best fit was used to characterize the stiffness of each zone. Both the loading and unloading curves were used for segmentation of the different shear zones. We note that at 40% strain, waveforms depart from pure sinusoidal waveforms, limiting their comparison for phase shift. (B) Comparison of 10% and 40% strain loading curves. Through pilot studies, 10% strain was found to not significantly enter the CZ or TZ.

**Table S1:** Age matched patient demographics. Total number of individuals, discs collected, and outer annulus fibrosus (OAF) tissue in each condition. * *p* < 0.05. (^†^) Due to an applied filter from a new 10 N Bose load cell, 7 non‐DD cubes were not included in dynamic mechanical analysis (*N* = 6). (—) denotes data that is unavailable.

**Table S2:** Summary of data distribution and statistical tests performed. Shapiro–Wilk tests were conducted using R within Visual Studio Code. Parametric statistics tests were applied to comparison groups with normal distribution data (*p* > 0.05), while non‐parametric statistics tests were used for groups with non‐normal distributions (*p* < 0.05). (*) denotes groups that were non‐normally distributed and analyzed using non‐parametric methods.

**Table S3:** Age matched summary of mechanical data collected from non‐DD and DD individuals in the radial (G1) and circumferential (G2) orientation. Values are presented as mean ± standard deviation. * *p* < 0.05 increased compared to the other group using Mann Whitney U test.^†^ *p* < 0.05 comparison within group using Friedman test.^a b c^ *p* < 0.05 increased compared to ranked values respective of increasing order using post hoc Mann Whitney U test.

**Table S4:** Summary of mechanical data collected from degenerative disc disease (DD) and non‐DD individuals. Sex comparison between female (F) and male (M) tissue. Values are presented as mean ± standard deviation. (*) denotes *p* < 0.05 increased compared to other sex.

**Table S5:** 40% large cohort summary of mechanical data collected from non‐DD and DD individuals in the radial (G1) and circumferential (G2) orientation. Values are presented as mean ± standard deviation. (*) denotes *p* < 0.05 increased compared to the other group using Mann Whitney U test. (^†^) denotes *p* < 0.05 comparison within group using Friedman test. (a b c) denotes statistically significant increases (*p* < 0.05) compared to ranked values in increasing order using post hoc test.

**Table S6:** Shear modulus variability between adjacent cubes from one annulus fibrosus (AF) tissue. Two cubes from each tissue were sectioned adjacently to each other and tested using the same parameters and in the same orientation (radial (G1)). Cube 2 were always subsequently after cube 1. Differences between adjacent cubes was averaged for intradiscal shear modulus variability.

**Video S1:** Dynamic mechanical analysis (DMA) of annulus fibrosus (AF) tissue. AF samples (white tissue with blue markings) were adhered to spring‐loaded aluminum plates (silver rectangles) using cyanoacrylate gel (Gorilla Superglue; The Gorilla Glue Company, Ohio, USA). The plates were mounted in acrylic clamps (black L‐shaped), which were attached to either a Bose 3220 or 3230 test frame with a 10 N Bose load cell (not shown). The plates were adjusted to create a centered 4.5 mm gap. The recording shows the DMA protocol, with the test frame applying ±0.5 mm displacement at 1 Hz while mist was applied to the tissue. Video recordings were used to verify proper tissue adhesion to each plate before initiating data collection.

## Data Availability

The data that support the findings of this study are available on request from the corresponding author. The data are not publicly available due to privacy or ethical restrictions.
