# Supplementary figures and images for: Reduced Shear Modulus and Altered Lamellar Morphology of the Outer Annulus Fibrosus in Painful Intervertebral Disc Degeneration Compared With Tissue From Non‐Surgical Controls
Source: JOR Spine. 2025 Oct 8;8(4):e70123. doi: 10.1002/jsp2.70123 (PMC12507480; doi:10.1002/jsp2.70123)

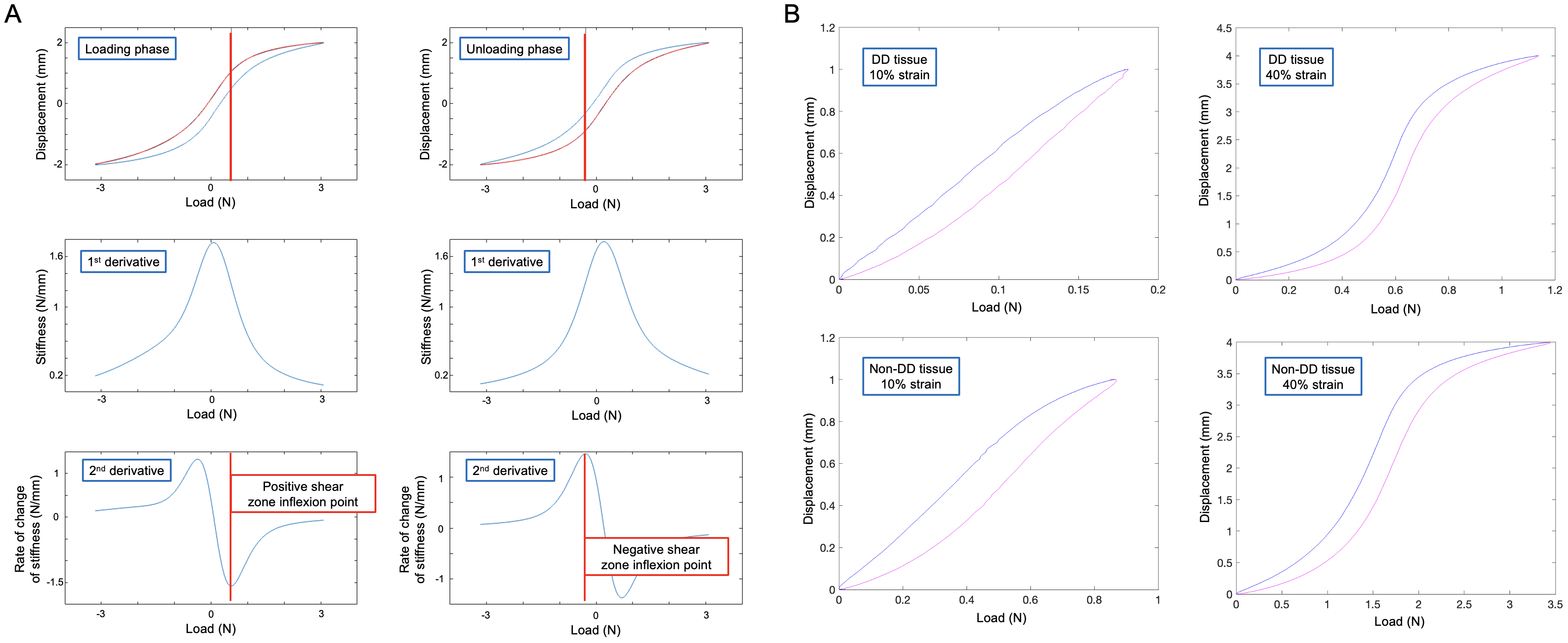

Supplement: Supplementary file 1 — Figure S1: Dynamic Mechanical Analysis (DMA) at 40% shear strain. (A) Neutral zone (NZ) calculation: To separate the curve into a compressive shear zone (CZ), tensile shear zone (TZ), and NZ, the borders of the NZ were determined using the maximum and minimum of the second derivative, which would note the change in shear compliance. A linear line of best fit was used to characterize the stiffness of each zone. Both the loading and unloading curves were used for segmentation of the different shear zones. We note that at 40% strain, waveforms depart from pure sinusoidal waveforms, limiting their comparison for phase shift. (B) Comparison of 10% and 40% strain loading curves. Through pilot studies, 10% strain was found to not significantly enter the CZ or TZ. [file JSP2-8-e70123-s003.tiff]
