# Supplementary material for: Reduced Shear Modulus and Altered Lamellar Morphology of the Outer Annulus Fibrosus in Painful Intervertebral Disc Degeneration Compared With Tissue From Non‐Surgical Controls
Source: JOR Spine. 2025 Oct 8;8(4):e70123. doi: 10.1002/jsp2.70123 (PMC12507480; doi:10.1002/jsp2.70123)
Supplement: Supplementary file 2 — Table S1: Age matched patient demographics. Total number of individuals, discs collected, and outer annulus fibrosus (OAF) tissue in each condition. * p < 0.05. (†) Due to an applied filter from a new 10 N Bose load cell, 7 non‐DD cubes were not included in dynamic mechanical analysis (N = 6). (—) denotes data that is unavailable. [file JSP2-8-e70123-s006.docx]

*Table S1: Age matched patient demographics. Total number of individuals, discs collected, and outer annulus fibrosus (OAF) tissue in each condition. * p < 0.05. ^†^ Due to an applied filter from a new 10N Bose load cell, 7 non-DD cubes were not included in dynamic mechanical analysis (N = 6). (—) denotes data that is unavailable.*

|  |  | Non-DD | DD |
| --- | --- | --- | --- |
| Individuals | Included individuals | 7 | 13 |
|  | Age (years) | 37.6 ± 6.6 | 39.2 ± 6.7 |
|  | Sex (Female / Male) | 3 / 4 | 9 / 4 |
|  | BMI (kg/m^2^) | — | 25.6 ± 6.5 |
| Tissues | Total included discs (L4-5 / L5-S1) | 13 (7 / 6) | 13 (6 / 7) |
|  | OAF cubes tested (G23 / G13) | 26 (13 / 13 ^†^) | 26 (13 / 13) |
|  | Modified Pfirrmann Grade (1-8) | — | 6.5 ± 1.7 |
|  | Radiographic Degeneration Grade | 0.25 ± 0.45 | — |
