# Supplementary material for: Reduced Shear Modulus and Altered Lamellar Morphology of the Outer Annulus Fibrosus in Painful Intervertebral Disc Degeneration Compared With Tissue From Non‐Surgical Controls
Source: JOR Spine. 2025 Oct 8;8(4):e70123. doi: 10.1002/jsp2.70123 (PMC12507480; doi:10.1002/jsp2.70123)
Supplement: Supplementary file 3 — Table S2: Summary of data distribution and statistical tests performed. Shapiro–Wilk tests were conducted using R within Visual Studio Code. Parametric statistics tests were applied to comparison groups with normal distribution data (p > 0.05), while non‐parametric statistics tests were used for groups with non‐normal distributions (p < 0.05). (*) denotes groups that were non‐normally distributed and analyzed using non‐parametric methods. [file JSP2-8-e70123-s001.docx]

*Table S2: Summary of data distribution and statistical tests performed. Shapiro-Wilk tests were conducted using R within Visual Studio Code. Parametric statistics tests were applied to comparison groups with normal distribution data (p > 0.05), while non-parametric statistics tests were used for groups with non-normal distributions (p < 0.05). ^*^ denotes groups that were non-normally distributed and analyzed using non-parametric methods.*

|  |  | Radial (G1) | | Circ (G2) | |
| --- | --- | --- | --- | --- | --- |
|  |  | Non-DD | DD | Non-DD | DD |
| Shear modulus | 10% strain | p = 0.264 | p < 0.001 * | p = 0.060 | p = 0.067 |
| Stress relaxation | 2.5% strain | p = 0.418 | p = 0.018 * | p = 0.784 | p = 0.014 * |
|  | 5% strain | p = 0.827 | p = 0.018 * | p = 0.569 | p = 0.003 * |
|  | 7.5% strain | p = 0.846 | p = 0.015 * | p = 0.945 | p = 0.006 * |
|  | 10% strain | p = 0.394 | p = 0.008 * | p = 0.755 | p = 0.044 * |
| Hysteresis | 0.01 Hz | p = 0.521 | p = 0.035 * | p = 0.099 | p = 0.009 * |
|  | 0.1 Hz | p = 0.400 | p = 0.070 | p = 0.055 | p = 0.007 * |
|  | 1 Hz | p = 0.804 | p = 0.119 | p = 0.06 | p = 0.007 * |
| Tan (δ) | 0.01 Hz | p = 0.023 * | p = 0.150 | p = 0.259 | p = 0.017 * |
|  | 0.1 Hz | p = 0.049 * | p = 0.596 | p = 0.866 | p = 0.894 |
|  | 1 Hz | p = 0.213 | p = 0.245 | p = 0.069 | p = 0.819 |
| \|G*\| | 0.01 Hz | p = 0.994 | p = 0.012 * | p = 0.334 | p = 0.037 * |
|  | 0.1 Hz | p = 0.998 | p = 0.013 * | p = 0.377 | p = 0.042 * |
|  | 1 Hz | p = 0.985 | p = 0.018 * | p = 0.251 | p = 0.052 |
