# Supplementary material for: Reduced Shear Modulus and Altered Lamellar Morphology of the Outer Annulus Fibrosus in Painful Intervertebral Disc Degeneration Compared With Tissue From Non‐Surgical Controls
Source: JOR Spine. 2025 Oct 8;8(4):e70123. doi: 10.1002/jsp2.70123 (PMC12507480; doi:10.1002/jsp2.70123)
Supplement: Supplementary file 4 — Table S3: Age matched summary of mechanical data collected from non‐DD and DD individuals in the radial (G1) and circumferential (G2) orientation. Values are presented as mean ± standard deviation. * p < 0.05 increased compared to the other group using Mann Whitney U test.† p < 0.05 comparison within group using Friedman test.a b c p < 0.05 increased compared to ranked values respective of increasing order using post hoc Mann Whitney U test. [file JSP2-8-e70123-s008.docx]

*Table S3: Age matched summary of mechanical data collected from non-DD and DD individuals in the radial (G1) and circumferential (G2) orientation. Values are presented as mean ± standard deviation. * p < 0.05 increased compared to the other group using Mann Whitney U test. ^†^ p < 0.05 comparison within group using Friedman test. ^a b c^ p < 0.05 increased compared to ranked values respective of increasing order using post-hoc Mann Whitney U test.*

|  |  | Radial (G1) | | Circ (G2) | |
| --- | --- | --- | --- | --- | --- |
|  |  | Non-DD | DD | Non-DD | DD |
| Shear modulus (kPa) | 10% strain | 83.0 ± 40.0 * | 21.8 ± 24.7 | 226.2 ± 79.4 * ^†^ | 43.0 ± 38.4 |
| Shear modulus (kPa) | 40% strain | 143.4 ± 57.5 * | 30.6 ± 22.8 | 325.2 ± 153.6 * ^†^ | 52.7 ± 42.1 |
| 40% stress relaxation (kPa) | 10% strain | 3.6 ± 1.4 * ^†^ | 1.4 ± 1.3 ^†^ | 8.4 ± 3.1 ^* †^ | 2.6 ± 2.7 ^†^ |
|  | 20% strain | 5.3 ± 2 * ^† a^ | 2.1 ± 1.6 ^† a^ | 12 ± 4 * ^† a^ | 3.9 ± 3.3 ^† a^ |
|  | 30% strain | 7.8 ± 2.6 * ^† a b^ | 2.9 ± 2 ^† a b^ | 18.1 ± 5.9 * ^† a b^ | 5.8 ± 3.9 ^† a b^ |
|  | 40% strain | 13.4 ± 4.2 * ^† a b c^ | 4.6 ± 2.4 ^† a b c^ | 32.4 ± 12.5 * ^† a b c^ | 10 ± 7.1 ^† a b c^ |
| Hysteresis (%) | 0.01Hz | 30.2 ± 9.5 ^† b c^ | 47.22 ± 8.9 ^† b c^ | 35.7 ± 10.8 | 42.89 ± 10.7 |
|  | 0.1Hz | 27.1 ± 8.3 ^†^ | 39.7 ± 8.6 ^†^ | 30 ± 5 | 37.39 ± 7 |
|  | 1Hz | 27.6 ± 8.1 ^†^ | 39.48 ± 7.9 ^†^ | 31.6 ± 5.5 | 38.21 ± 7.4 |
| Hysteresis (µJoules) | 0.01Hz | 93.47 ± 25.2 * | 40.07 ± 34.5 ^†^ | 201.45 ± 89.5 * | 55.74 ± 55.4 ^†^ |
|  | 0.1Hz | 95.87 ± 26 * ^†^ | 41.26 ± 33.2 ^†^ | 190.73 ± 54.2 * | 58.28 ± 53.8 ^†^ |
|  | 1Hz | 108.6 ± 27.8 * ^† a b^ | 48.1 ± 37.7 ^† a b^ | 218.62 ± 51.8 * | 70.91 ± 67.4 ^† a b^ |
| Tan (δ) | 0.01Hz | 0.18 ± 0.1 ^† b c^ | 0.26 ± 0 * ^† b c^ | 0.18 ± 0 | 0.24 ± 0.1 * ^† b c^ |
|  | 0.1Hz | 0.16 ± 0.1 ^†^ | 0.23 ± 0 * ^†^ | 0.17 ± 0 | 0.21 ± 0 ^†^ |
|  | 1Hz | 0.16 ± 0.1 ^†^ | 0.22 ± 0.1 * ^†^ | 0.18 ± 0 | 0.21 ± 0 ^†^ |
| G’ (kPa) | 0.01Hz | 137.4 ± 46.5 * ^†^ | 48.4 ± 40.7 ^†^ | 313.4 ± 112.2 * ^†^ | 84.8 ± 68.9 ^†^ |
|  | 0.1Hz | 156.5 ± 49.8 * ^† a^ | 55.8 ± 45.2 ^† a^ | 335.4 ± 107.1 * ^†^ | 96.3 ± 75.5 ^† a^ |
|  | 1Hz | 175.5 ± 54.5 * ^† a b^ | 65 ± 50.8 ^† a b^ | 368.9 ± 112.1 * ^†^ | 109.4 ± 82.8 ^† a b^ |
| G’’ (kPa) | 0.01Hz | 24.8 ± 8.5 * ^†^ | 11.7 ± 8.5 | 66.9 ± 35 * ^†^ | 18.5 ± 13.8 ^†^ |
|  | 0.1Hz | 25.6 ± 8.3 * ^†^ | 11.8 ± 8.2 | 60.5 ± 21.5 * ^†^ | 18.7 ± 13.3 ^†^ |
|  | 1Hz | 29.5 ± 9.4 * ^† a b^ | 13.9 ± 9.3 | 68.2 ± 19.9 * ^†^ | 22.2 ± 15.5 ^† a b^ |
| \|G*\| (kPa) | 0.01Hz | 139.8 ± 46.8 * ^†^ | 44.43 ± 42 ^†^ | 320.7 ± 116.8 * ^†^ | 75.24 ± 82.6 ^†^ |
|  | 0.1Hz | 158.7 ± 50.1 * ^† a^ | 51.02 ± 46.6 ^† a^ | 340.9 ± 108.8 * ^†^ | 86.47 ± 90.9 ^† a^ |
|  | 1Hz | 178.1 ± 54.9 * ^† a b^ | 59.65 ± 52.6 ^† a b^ | 375.3 ± 113.3 * ^†^ | 98.94 ± 99.9 ^† a b^ |
| NZ length (mm) | 1Hz | 1.9 ± 0.3 | 2.0 ± 0.3 | 1.9 ± 0.6 | 2.3 ± 0.5 |
| NZ stiffness (Nmm) | 1Hz | 0.5 ± 0.2 * | 0.1 ± 0.1 | 0.8 ± 0.3 * ^†^ | 0.2 ± 0.3 |
| CZ stiffness (Nmm) | 1Hz | 1.9 ± 0.8 * | 0.6 ± 0.4 | 3.9 ± 2.3 * ^†^ | 1.2 ± 0.7 ^†^ |
| TZ stiffness (Nmm) | 1Hz | 1.4 ± 0.5 * | 0.4 ± 0.2 | 2.2 ± 1.3 * ^†^ | 0.7 ± 0.4 ^†^ |
