# Supplementary material for: Reduced Shear Modulus and Altered Lamellar Morphology of the Outer Annulus Fibrosus in Painful Intervertebral Disc Degeneration Compared With Tissue From Non‐Surgical Controls
Source: JOR Spine. 2025 Oct 8;8(4):e70123. doi: 10.1002/jsp2.70123 (PMC12507480; doi:10.1002/jsp2.70123)
Supplement: Supplementary file 5 — Table S4: Summary of mechanical data collected from degenerative disc disease (DD) and non‐DD individuals. Sex comparison between female (F) and male (M) tissue. Values are presented as mean ± standard deviation. (*) denotes p < 0.05 increased compared to other sex. [file JSP2-8-e70123-s002.docx]

*Table S4: Summary of mechanical data collected from degenerative disc disease (DD) and non-DD individuals. Sex comparison between female (F) and male (M) tissue. Values are presented as mean ± standard deviation. ^*^ denotes p < 0.05 increased compared to other sex.*

|  |  | Radial (G1) | | | | Circ (G2) | | | | |
| --- | --- | --- | --- | --- | --- | --- | --- | --- | --- | --- |
|  |  | Non-DD | | DD | | Non-DD | | DD | | |
|  |  | F | M | F | M | F | M | | F | M |
| Shear modulus (kPa) | 10% | 111.6 ± 27.0 (p = 0.07) | 65.2 ± 39.5 | 25.7 ± 24.6 | 20.7 ± 21.4 | 260.7 ± 105.8  (p = 0.16) | 204.6 ± 60.7 | 53.5 ± 44.5 | | 48.1 ± 21.1 |
| Stress relaxation (kPa) | 2.5% | 2.3 ± 0.6 | 3.1 ± 1.4 | 1.0 ± 0.8 | 1.5 ± 1.5 | 4.0 ± 2.1 | 6.4 ± 1.9 | 2.0 ± 1.7 | | 0.8 ± 0.3 |
|  | 5% | 2.7 ± 0.6 | 3.5 ± 1.4 | 1.1 ± 0.8 | 1.6 ± 1.5 | 4.5 ± 2.1 | 7.5 ± 1.9 | 2.3 ± 2.2 | | 1.0 ± 0.4 |
|  | 7.5% | 3.2 ± 0.8 | 3.7 ± 1.4 | 1.1 ± 0.8 | 1.7 ± 1.7 | 5.4 ± 2.5 | 8.2 ± 2.3 | 2.4 ± 2.2 | | 1.3 ± 0.5 |
|  | 10% | 3.7 ± 0.9 | 4.0 ± 1.6 | 1.1 ± 0.7 | 1.9 ± 1.7 | 6.7 ± 3.0 | 9.0 ± 2.2 | 2.7 ± 2.5 | | 1.7 ± 0.9 |
| Hysteresis (µJoules) | 0.01 Hz | 96.7 ± 21.1 | 91.5 ± 28.6 | 42.6 ± 27.6 | 49.8 ± 44.1 | 139.6 ± 10.6 | 232.4 ± 97.3 | 72.9 ± 55.7 | | 42.9 ± 20.4 |
|  | 0.1 Hz | 100.6 ± 21.3 | 92.9 ± 29.5 | 44.4 ± 27.7 | 49.2 ± 42.9 | 159.2 ± 11.7 | 206.5 ± 62.1 | 75.8 ± 56.6 | | 45.8 ± 18.1 |
|  | 1 Hz | 116.4 ± 22.8 | 103.7 ± 30.9 | 51.9 ± 31.6 | 53.8 ± 43.6 | 191.6 ± 15.7 | 232.1 ± 60.4 | 90.6 ± 67.7 | | 51.9 ± 20.2 |
| Tan (δ) | 0.01 Hz | 0.17 ± 0.05 | 0.19 ± 0.05 | 0.27 ± 0.07 | 0.26 ± 0.06 | 0.17 ± 0.01 | 0.18 ± 0.02 | 0.24 ± 0.06 | | 0.2 ± 0.03 |
|  | 0.1 Hz | 0.15 ± 0.05 | 0.16 ± 0.06 | 0.24 ± 0.06 | 0.21 ± 0.06 | 0.17 ± 0 | 0.17 ± 0.04 | 0.21 ± 0.04 | | 0.18 ± 0.04 |
|  | 1 Hz | 0.15 ± 0.05 | 0.16 ± 0.06 | 0.23 ± 0.07 | 0.2 ± 0.07 | 0.19 ± 0 | 0.17 ± 0.04 | 0.22 ± 0.05 | | 0.18 ± 0.05 |
| \|G*\| (kPa) | 0.01 Hz | 154.8 ± 45.5 | 130.4 ± 48.1 | 50.3 ± 39.5 | 49 ± 47.6 | 241.4 ± 46.5 | 360.3 ± 125.4 | 95.6 ± 80.9 | | 69.4 ± 40.3 |
|  | 0.1 Hz | 172.3 ± 46.9 | 150.1 ± 53.2 | 57.5 ± 43.7 | 56.4 ± 52.6 | 263.8 ± 60.2 | 379.5 ± 112.1 | 107.2 ± 88.4 | | 80.3 ± 44.3 |
|  | 1 Hz | 194.3 ± 49.8 | 169.0 ± 58.6 | 67.2 ± 49.3 | 65.6 ± 58.7 | 296.7 ± 63.4 | 414.6 ± 117.8 | 122.1 ± 97.2 | | 91.2 ± 47.6 |
