# Supplementary material for: Reduced Shear Modulus and Altered Lamellar Morphology of the Outer Annulus Fibrosus in Painful Intervertebral Disc Degeneration Compared With Tissue From Non‐Surgical Controls
Source: JOR Spine. 2025 Oct 8;8(4):e70123. doi: 10.1002/jsp2.70123 (PMC12507480; doi:10.1002/jsp2.70123)
Supplement: Supplementary file 6 — Table S5: 40% large cohort summary of mechanical data collected from non‐DD and DD individuals in the radial (G1) and circumferential (G2) orientation. Values are presented as mean ± standard deviation. (*) denotes p < 0.05 increased compared to the other group using Mann Whitney U test. (†) denotes p < 0.05 comparison within group using Friedman test. (a b c) denotes statistically significant increases (p < 0.05) compared to ranked values in increasing order using post hoc test. [file JSP2-8-e70123-s004.docx]

*Table S5: 40% large cohort summary of mechanical data collected from non-DD and DD individuals in the radial (G1) and circumferential (G2) orientation. Values are presented as mean ± standard deviation. ^*^ denotes p < 0.05 increased compared to the other group using Mann Whitney U test. ^†^ denotes p < 0.05 comparison within group using Friedman test. ^a b c^ denotes statistically significant increases (p < 0.05) compared to ranked values in increasing order using post-hoc test.*

|  |  | Radial (G1) | | Circ (G2) | |
| --- | --- | --- | --- | --- | --- |
|  |  | Non-DD | DD | Non-DD | DD |
| Shear modulus (kPa) | 40% strain | 143.4 ± 59.3 * | 33.2 ± 24.1 | 301.1 ± 153.4 * ^†^ | 63.1 ± 46.6 ^†^ |
| 40% stress relaxation (kPa) | 10% strain | 3.6 ± 1.4 * ^†^ | 1.8 ± 1.7 ^†^ | 8.4 ± 3.1 * ^†^ | 3 ± 2.7 ^†^ |
|  | 20% strain | 5.3 ± 2 * ^† a^ | 2.6 ± 2.3 ^† a^ | 12 ± 4 * ^† a^ | 4.3 ± 3.2 ^† a^ |
|  | 30% strain | 7.8 ± 2.6 * ^† a b^ | 3.5 ± 2.6 ^† a b^ | 18.1 ± 5.9 * ^† a b^ | 6.3 ± 3.9 ^† a b^ |
|  | 40% strain | 13.4 ± 4.2 * ^† a b c^ | 5.3 ± 3 ^† a b c^ | 32.4 ± 12.5 * ^† a b c^ | 10.2 ± 6.3 ^† a b c^ |
| Hysteresis (µJoules) | 0.01 Hz | 1148.7 ± 318.6 | 499.5 ± 319.8 | 2770.8 ± 1074.8 ^†^ | 725.6 ± 455.6 |
|  | 0.1 Hz | 1175.7 ± 300.2 | 512.9 ± 291.6 | 2835.2 ± 1305.6 ^†^ | 710.6 ± 408.0 |
|  | 1 Hz | 1348.8 ± 332.1 | 601.2 ± 322.7 | 3043.0 ± 1463.4 ^†^ | 829.5 ± 463.5 |
| Tan (δ) | 0.01 Hz | 0.14 ± 0 ^†^ | 0.22 ± 0.1 | 0.16 ± 0 ^†^ | 0.19 ± 0.1 |
|  | 0.1 Hz | 0.12 ± 0 ^†^ | 0.2 ± 0.1 | 0.2 ± 0.1 ^†^ | 0.18 ± 0.1 |
|  | 1 Hz | 0.13 ± 0 ^†^ | 0.22 ± 0.1 | 0.21 ± 0.1 ^†^ | 0.19 ± 0.1 |
| \|G*\| (kPa) | 0.01 Hz | 173.26 ± 56.5 | 51.24 ± 36.7 | 313.64 ± 123.2 | 85.57 ± 53 |
|  | 0.1 Hz | 194.02 ± 62.2 | 58.96 ± 40.6 | 334.9 ± 143.2 | 96.46 ± 57.8 |
|  | 1 Hz | 207.57 ± 64.4 | 65.89 ± 44.3 | 352.43 ± 142.6 | 107.09 ± 62.2 |
| NZ length (mm) | 1 Hz | 1.9 ± 0.3 | 1.9 ± 0.4 | 1.9 ± 0.6 | 2.3 ± 0.5 |
| NZ stiffness (Nmm) | 1 Hz | 0.5 ± 0.2 * | 0.2 ± 0.1 | 0.8 ± 0.3 * ^†^ | 0.3 ± 0.3 |
| CZ stiffness (Nmm) | 1 Hz | 1.9 ± 0.8 * | 0.7 ± 0.3 | 3.9 ± 2.3 * ^†^ | 1.2 ± 0.7 ^†^ |
| TZ stiffness (Nmm) | 1 Hz | 1.4 ± 0.5 * | 0.4 ± 0.2 | 2.2 ± 1.3 * ^†^ | 0.7 ± 0.5 * |
