# Supplementary material for: Reduced Shear Modulus and Altered Lamellar Morphology of the Outer Annulus Fibrosus in Painful Intervertebral Disc Degeneration Compared With Tissue From Non‐Surgical Controls
Source: JOR Spine. 2025 Oct 8;8(4):e70123. doi: 10.1002/jsp2.70123 (PMC12507480; doi:10.1002/jsp2.70123)
Supplement: Supplementary file 7 — Table S6: Shear modulus variability between adjacent cubes from one annulus fibrosus (AF) tissue. Two cubes from each tissue were sectioned adjacently to each other and tested using the same parameters and in the same orientation (radial (G1)). Cube 2 were always subsequently after cube 1. Differences between adjacent cubes was averaged for intradiscal shear modulus variability. [file JSP2-8-e70123-s007.docx]

*Table S6: Shear modulus variability between adjacent cubes from one annulus fibrosus (AF) tissue. Two cubes from each tissue were sectioned adjacently to each other and tested using the same parameters and in the same orientation (radial (G1)). Cube 2 were always subsequently after cube 1. Differences between adjacent cubes was averaged for intradiscal shear modulus variability.*

|  | AF 1 | AF 2 | AF 3 | AF 4 | AF 5 | AF 6 | Average |
| --- | --- | --- | --- | --- | --- | --- | --- |
| Cube 1 | 71.6 | 14.8 | 20.2 | 3.7 | 7.9 | 2.5 | 20.1 ± 26.0 |
| Cube 2 | 49.3 | 12.4 | 20.9 | 0.8 | 6.9 | 1.1 | 15.2 ± 18.3 |
| Difference | 22.3 | 2.4 | 0.7 | 2.9 | 1.0 | 1.4 | 4.9 ± 8.6 |
